# Supporting engagement, behavior change, and equitable self-management in menstrual and menopausal health: A multi-agent digital-health framework

**DOI:** 10.1371/journal.pdig.0001821

**Published:** 2026-11-05

**Authors:** Koichiro Yuji, Wakako Yuji, Maika Nariai, Yuki Enomoto, Osamu Wada-Hiraike

**Affiliations:** 1 Project Division of International Healthcare Innovation Research, The Institute of Medical Science, the University of Tokyo, Tokyo, Japan; 2 Department of Research, Kitasato Institute Hospital, Tokyo, Japan; 3 Department of Obstetrics and Gynecology, Graduate School of Medicine, The University of Tokyo, Tokyo, Japan; 4 Department of Obstetrics and Gynecology, Nippon Medical School Hospital, Tokyo, Japan; SCUT: South China University of Technology, CHINA

## Abstract

Menstrual-cycle–related conditions and, later, menopausal symptoms are highly prevalent across the female life course and impose a large, under-recognized burden on quality of life and work, yet digital tools to support self-management are fragmented, hard to sustain, and unevenly reached. We propose HERA (Health Engagement and Reflective Agent architecture), a conceptual, hypothesis-generating framework for supporting engagement, behavior change, and equitable self-management in menstrual and menopausal health across the life course. Under human governance, HERA combines two adaptive agents (interpretation and orchestration) with three rule-based modules (self-monitoring and data integration, guidance and content governance, and equity and engagement monitoring) that integrate self-monitoring, deliver personalized feedback, surface governed guidance, monitor equity, and coordinate hand-offs to clinicians and occupational-health services. Distinct from clinician-facing diagnostic agents, HERA is patient-facing and treats equity and engagement as first-class. We distinguish technically feasible from aspirational elements and set out a staged, threshold-gated evaluation plan. HERA is a design reference, not a validated system.

Menstrual-cycle–related conditions and, later, the menopause transition are common yet under-supported. A nationwide survey of over 32,000 women estimated roughly 9 days of presenteeism-related productivity loss per year from menstrual symptoms [1], and menopausal symptoms can affect quality of life and work [2].

Period-tracking apps are widely used and wearables are emerging [3,4], and public-health policy emphasizes personal-health-record infrastructure, inclusion, and a life course approach [5]. Yet engagement is hard to sustain, content quality varies, and disparities in access and digital literacy risk a widening divide.

Agentic AI—autonomous software that plans toward goals across steps, requiring governance and human oversight [6]—has been proposed for clinical triage [7] and, in our own work, laboratory medicine [8]; these are professional-facing and diagnostic. Menstrual and menopausal health poses a complementary, patient-facing problem: sustained self-monitoring, behavior change, equitable engagement, and follow-through across settings.

We propose HERA (Health Engagement and Reflective Agent architecture), a conceptual, hypothesis-generating framework—not an implemented or validated system—for menstrual and menopausal self-management. HERA combines two adaptive agents with three rule-based modules under human governance (Fig 1). The interpretation agent chooses among behavior change techniques [9], such as goal-setting or prompts, or defers, according to recent adherence and signal confidence; the orchestration agent reconciles outputs, manages consent, and escalates when thresholds are crossed. Where components disagree, it defers to the more conservative output and logs the conflict. The rule-based modules integrate data the user chooses to share (wearable signals; symptom and cycle logs; patient-reported outcomes; consented records), govern curated guidance, and monitor equity. Components communicate through the orchestration layer; under high uncertainty, automated suggestions are suppressed and escalated for human review. All outputs are advisory and state their uncertainty plainly, since users may read them as medical advice; the individual and their clinicians retain final authority.

**Fig 1:**
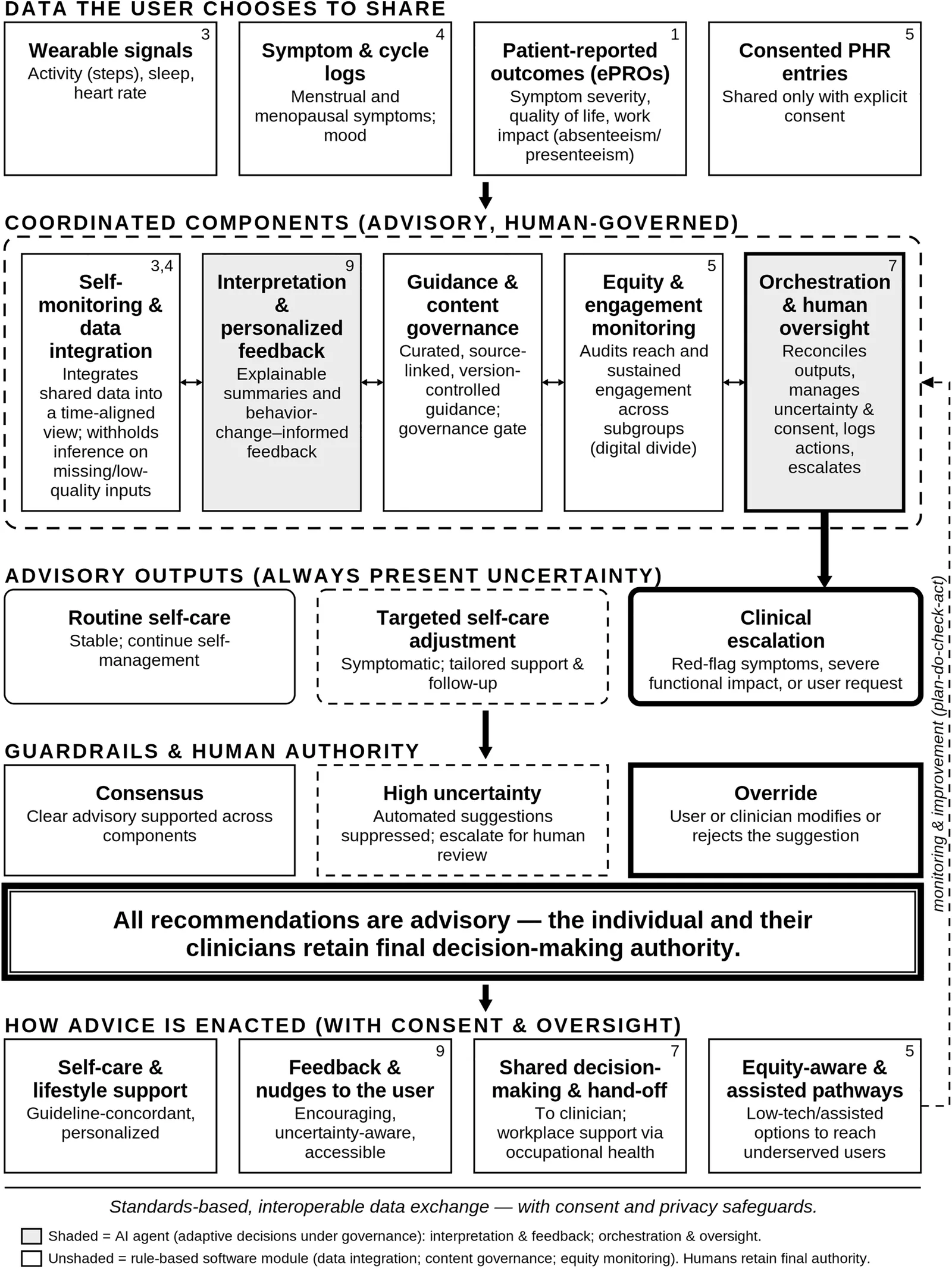
HERA multi-agent framework (conceptual). Two adaptive agents (shaded: interpretation; orchestration) and three rule-based modules (data integration; content governance; equity and engagement monitoring) operate under human governance on shared data to support menstrual and menopausal self-management across the life course.

A worked example marks where self-management ends and clinical care begins. A user who logs increasingly heavy menstrual bleeding with worsening daily function moves from routine self-care to targeted adjustment with tailored support; when red-flag thresholds are crossed, the orchestration agent withholds self-management advice and directs the user to clinical assessment. Occupational-health services may separately support workplace accommodations but are not a route for assessing bleeding. Menstrual and menopausal use cases differ in trajectories, risks, and pathways, so thresholds would be set separately.

Two choices distinguish HERA. First, sustained engagement is a first-class objective and evaluation target, since many users disengage early [10]. Second, equity is architectural. The equity module reports reach and engagement across subgroups and flags gaps to human operators, who can add assisted or low-technology pathways, language adaptations, and outreach; correction is human-flagged, not automated. Because designs that assume a cisgender, heterosexual user can exclude marginalized groups, capability-sensitive design and co-design with intended users are part of the development pathway [11]. One author, an intended user herself, contributed to the framework; broader co-design remains necessary. Although anchored in one national policy context [5], the architecture is meant to be portable rather than transplanted: in lower-resourced settings, the equity module would prioritize low-technology channels—messaging-based entry or community-health-worker mediation—with locally governed guidance, co-design, and validation.

Technically feasible now are integration of wearable and app data with patient-reported outcomes, interpretable feedback, version-controlled guidance under human oversight, and subgroup engagement audits—though scaling individualized behavior change support remains hard. Aspirational, requiring research, validation, and governance, are continuous population-level monitoring, fully dynamic guidance retrieval, and automated resolution of disagreement without human review.

Existing menstrual and menopausal tools largely track cycles and symptoms [12]; a generic health-agent framework is domain-agnostic [13], an equity-by-design chatbot roadmap offers design stages rather than an architecture [14], and agentic AI in healthcare remains largely exploratory, with few studies involving patients [15]. These cannot simply be adapted, because each lacks the longitudinal state linking adolescence to the menopause transition, and none carries an equity signal into its routing decisions. To our knowledge, none integrates life course equity and engagement monitoring with self-monitoring, governed guidance, and clinician and occupational-health oversight for this domain (S1 Text).

Because HERA would combine reproductive, wearable, clinical, and employment-related data, user choice over sharing is not sufficient. Governance must address retention, secondary use, inferred information, and role-based access, and must separate the occupational-health handoff from any employer access. The escalation function resembles clinical decision support and would fall under the corresponding medical-device and health-data regulation, with transparent logging of AI-influenced actions, defined update procedures, and responsibility for advisory outputs and overrides.

A minimum indicator set spans equitable reach and retention, behavioral and functional outcomes (symptom scores, quality of life, and work productivity), and safety measures (appropriate-escalation, over-referral, human-override, and missed red-flag rates). Thresholds should be prespecified and justified for the study setting. Illustratively, the equity gate would require 12-week retention in the least-reached subgroup to be at least 0.8 times that in the best-reached subgroup (echoing the four-fifths convention), with an overall retention target such as 50%. Evidence should accrue in stages: retrospective baselining before HERA is built, then usability testing, a pilot, and controlled comparisons—against usual care (testing the whole package) and against a version with identical content, features, and human support but no agent coordination (isolating the architecture's contribution)—each gated on equity and safety thresholds (S1 Text).

Menstrual and menopausal health may be better supported when self-monitoring, feedback, guidance, and follow-through are integrated across the life course. Data gathered this way could also help clarify how the menstrual cycle affects other body systems. Agentic AI could help, but this is unproven: implementation, real-world usability, validation, clinical-outcome, and cost-effectiveness data are lacking, and benefit depends on governance, equity safeguards, and human oversight. We offer HERA openly for others to adapt, contest, and evaluate; showing that it is useful, safe, and equitable will take staged, transparent evidence.

## Supporting information

S1 Text Comparison of HERA with existing approaches (Table A) and staged evaluation plan (Table B). (DOCX)

## References

[1] Schoep ME, Adang EMM, Maas JWM, De Bie B, Aarts JWM, Nieboer TE. Productivity loss due to menstruation-related symptoms: a nationwide cross-sectional survey among 32 748 women. BMJ Open. 2019;9(6):e026186. doi: 10.1136/bmjopen-2018-026186 31248919 PMC6597634

[2] Hickey M, LaCroix AZ, Doust J, Mishra GD, Sivakami M, Garlick D, et al. An empowerment model for managing menopause. Lancet. 2024;403(10430):947–57. doi: 10.1016/S0140-6736(23)02799-X 38458214

[3] Lyzwinski L, Elgendi M, Menon C. Innovative approaches to menstruation and fertility tracking using wearable reproductive health technology: systematic review. J Med Internet Res. 2024;26:e45139. doi: 10.2196/45139 38358798 PMC10905339

[4] Broad A, Biswakarma R, Harper JC. A survey of women’s experiences of using period tracker applications: attitudes, ovulation prediction and how the accuracy of the app in predicting period start dates affects their feelings and behaviours. Womens Health (Lond). 2022;18:17455057221095246. doi: 10.1177/17455057221095246 35465788 PMC9047811

[5] Ministry of Health, Labour and Welfare (Japan). Kenkō Nippon 21 (third term) [Health Japan 21 (third term)]. Tokyo: MHLW; Available from: https://www.mhlw.go.jp/stf/seisakunitsuite/bunya/kenkou_iryou/kenkou/kenkounippon21_00006.html

[6] Karunanayake N. Next-generation agentic AI for transforming healthcare. Inform Health. 2025;2(2):73–83. doi: 10.1016/j.infoh.2025.03.001

[7] Njei B, Kanmounye US. A conceptual agentic AI architecture for MASLD-associated significant fibrosis in primary care. PLOS Digit Health. 2026;5(6):e0001500. doi: 10.1371/journal.pdig.0001500 42348555 PMC13298771

[8] Yuji K, Yuji W. Artificial general intelligence and the clinical laboratory: a paradigm shift toward Lab 2.0. Clin Chem Lab Med. 2026;64(11):2382–8. doi: 10.1515/cclm-2026-0789 42402007

[9] Michie S, Richardson M, Johnston M, Abraham C, Francis J, Hardeman W, et al. The behavior change technique taxonomy (v1) of 93 hierarchically clustered techniques: building an international consensus for the reporting of behavior change interventions. Ann Behav Med. 2013;46(1):81–95. doi: 10.1007/s12160-013-9486-6 23512568

[10] Amagai S, Pila S, Kaat AJ, Nowinski CJ, Gershon RC. Challenges in participant engagement and retention using mobile health apps: literature review. J Med Internet Res. 2022;24(4):e35120. doi: 10.2196/35120 35471414 PMC9092233

[11] Jacobs N, Evers J. Ethical perspectives on femtech: moving from concerns to capability-sensitive designs. Bioethics. 2023;37(5):430–9. doi: 10.1111/bioe.13148 36807364

[12] Johnson SC, O’Day J, Kraus E, Delp SL, Hicks JL. Decoding menstrual health across the lifespan: a scoping review of digital health tools in research. NPJ Womens Health. 2026;4(1):27. doi: 10.1038/s44294-026-00146-7 42712482 PMC13549953

[13] Abbasian M, Azimi I, Rahmani AM, Jain R. Conversational health agents: a personalized large language model-powered agent framework. JAMIA Open. 2025;8(4):ooaf067. doi: 10.1093/jamiaopen/ooaf067 40620478 PMC12228965

[14] Nadarzynski T, Knights N, Husbands D, Graham CA, Llewellyn CD, Buchanan T, et al. Achieving health equity through conversational AI: a roadmap for design and implementation of inclusive chatbots in healthcare. PLOS Digit Health. 2024;3(5):e0000492. doi: 10.1371/journal.pdig.0000492 38696359 PMC11065243

[15] Collaco BG, Haider SA, Prabha S, Gomez-Cabello CA, Genovese A, Wood NG, et al. The role of agentic artificial intelligence in healthcare: a scoping review. NPJ Digit Med. 2026;9(1):345. doi: 10.1038/s41746-026-02517-5 41832341 PMC13133135

