# Supplementary material for: Supporting engagement, behavior change, and equitable self-management in menstrual and menopausal health: A multi-agent digital-health framework
Source: PLOS Digit Health. 2026 Nov 5;5(11):e0001821. doi: 10.1371/journal.pdig.0001821 (PMC13739118; doi:10.1371/journal.pdig.0001821)
Supplement: Supplementary file 1 [file pdig.0001821.s001.docx]

**S1 Text. Comparison of HERA with existing approaches (Table A) and staged evaluation plan (Table B).**

*Supporting information for: Supporting engagement, behavior change, and equitable self-management in menstrual and menopausal health: a multi-agent digital-health framework.*

**Table A. HERA compared with the approaches cited in the main text, on the dimensions requested in review.**

| **Approach [ref]** | **Type of contribution** | **Primary users** | **Patient-facing functions** | **Data inputs** | **Governance** | **Escalation and hand-off** | **Clinician oversight** | **Equity monitoring** | **Clinical scope** |
| --- | --- | --- | --- | --- | --- | --- | --- | --- | --- |
| **Digital tools in menstrual and menopausal research [12]** | Scoping review of research using wearables and smartphone apps | Individuals tracking cycles and symptoms; researchers | Tracking of cycles, symptoms, and physiology | Physiological, behavioral, and symptom data from wearables and apps (e.g., basal body temperature, heart rate variability); urinary hormone tests | Privacy noted as a need for research planning and data handling | Not described | Not described | Differences in cycle characteristics across demographic groups reported; equity monitoring not described | Menstrual cycle and menopausal transition, across the reviewed studies |
| **openCHA [13]** | Open-source framework for LLM-powered conversational health agents | Users of conversational health agents | Personalized responses to health-related queries (demonstrated in diabetes management and food recommendation) | User health data, including wearable and mobile-health data; knowledge bases; AI and analysis tools | Privacy and security discussed | Not described | Not described | Barriers for underserved communities and model bias discussed; equity monitoring not described | Domain-agnostic |
| **Equity-by-design roadmap for health chatbots [14]** | Ten-stage roadmap for equitable design and implementation, from guideline analysis and stakeholder consultation | Designers and implementers of conversational AI in healthcare | Not applicable (design guidance) | Not applicable | Safety measures; service evaluation and auditing; maintenance; termination (roadmap stages) | Healthcare integration addressed as a design stage | Health professionals among the consulted stakeholders | Central aim; diversity and collaboration, co-production with communities | Not specific to a clinical domain |
| **ATLAS-Liver [7]** | Conceptual agentic reference architecture, not a deployed system | Primary-care clinicians (triage) | Primarily clinician-facing; patient-facing needs (explanation formats, communication preferences, support for lifestyle adherence) discussed | Routine electronic health record data | Governance-focused design | Structured hand-offs to clinicians; follow-through on testing and referral | Clinician-retained decision authority | Calibration and fairness monitoring; equity monitoring | Single condition (MASLD-associated fibrosis) |
| **Agentic AI in healthcare [15]** | Scoping review of agentic AI studies | Varied; most included studies were experimental, without patient recruitment | Coaching among the reported applications | Varied | Not the focus of the review | Not the focus of the review | Not the focus of the review | Not the focus of the review | Applications included cancer diagnosis, treatment planning, alert generation, coaching, and workflow optimization; most studies exploratory, one trial involving patients |
| **HERA (this article)** | Conceptual multi-agent framework, not an implemented or validated system | Individuals (patient-facing), linked to clinicians and occupational-health services | Behavior-change–informed feedback; curated, source-linked guidance; outputs state their uncertainty plainly | Wearable signals; symptom and cycle logs; patient-reported outcomes; consented personal-health-record entries; exchanged through standards-based interfaces (e.g., HL7 FHIR); values not comparable across devices are flagged rather than acted on | Retention, secondary use, inferred information, and role-based access; occupational-health handoff separated from employer access; escalation treated as clinical decision support; data security, including encryption and access control, specified at implementation; logging of AI-influenced actions, so that inappropriate or delayed advice can be traced and responsibility assigned in advance; defined updates | Red-flag thresholds drawn from clinical guidance, prespecified for each use case; red flags go to clinical assessment; occupational health for workplace support only | Clinicians retain final authority, approve escalation thresholds, and review cases under high uncertainty | Equity module reports reach and sustained engagement by subgroup; correction human-flagged; prespecified equity gate | Menstrual health through the menopause transition, with thresholds set separately for menstrual and menopausal use cases |

*Entries summarize what each cited source describes. “Not described” means that the source does not describe the feature; it does not imply that the feature is absent from every tool in the field. Reference numbers correspond to the main reference list. HL7 FHIR, Health Level Seven Fast Healthcare Interoperability Resources; LLM, large language model; MASLD, metabolic dysfunction-associated steatotic liver disease.*

**Table B. Staged evaluation plan: objective, primary outcome, and criterion for progression at each stage.**

| **Stage** | **Objective** | **Primary outcome** | **Safety and equity measures** | **Criterion for progression** |
| --- | --- | --- | --- | --- |
| **1. Retrospective baselining (before HERA is built)** | Characterize engagement, retention, and outcomes in existing cohorts and routine data for the intended setting | Baseline 12-week retention, overall and by subgroup | Baseline subgroup retention ratio; baseline rates of escalation-relevant events | Baseline quantified; thresholds for later stages prespecified and justified for the setting |
| **2. Usability testing** | Test usability and whether users understand that outputs are advisory and what their uncertainty means, across literacy and language groups | Usability on a validated measure, by subgroup | Misinterpretation of advisory outputs as medical advice; accessibility | No safety-critical misunderstanding; acceptable usability in every subgroup |
| **3. Pilot** | Assess feasibility, sustained engagement, and safety in routine use | 12-week retention (illustrative target 50%, to be justified for the setting) | Retention ratio of least- to best-reached subgroup of at least 0.8; appropriate escalation, over-referral, human override, missed red flags | Prespecified equity and safety thresholds met |
| **4a. Controlled comparison with usual care** | Test the whole intervention | Prespecified functional outcome for each use case (symptom scores, quality of life, or work productivity), over a prespecified follow-up of at least 12 weeks (about three menstrual cycles) and longer for menopausal use cases | As in stage 3 | Benefit without widening subgroup gaps; safety thresholds met |
| **4b. Controlled comparison with a non-agentic version** | Isolate the contribution of the architecture | As in 4a | As in stage 3 | Comparison version has identical content, features, and human support; the two agents are replaced by fixed rules (feedback on a predefined schedule, escalation on the same thresholds without adaptive reconciliation) |

*Sample size for each stage depends on the setting and the primary outcome chosen and is to be specified in the study protocol. All thresholds are illustrative and must be prespecified and justified for the setting in which HERA is evaluated.*
